# Supplementary material for: Gender Disparities in Vascular Access and One-Year Mortality among Incident Hemodialysis Patients: An Epidemiological Study in Lazio Region, Italy
Source: J Clin Med. 2021 Oct 30;10(21):5116. doi: 10.3390/jcm10215116 (PMC8584887; doi:10.3390/jcm10215116)
Supplement: Supplementary file 1 [file jcm-10-05116-s001.zip › jcm-1399964-supplementary.pdf]

Table S1. Distribution of socio-demographic, clinical, and welfare characteristics according to type of vascular access for hemodialysis initiation (AVF and CVC). Incident hemodialysis patients from RRDTL Lazio Region (Italy) 2008–2018.

		VASCULAR ACCESS							
		**AVF**			**CVC**			**Total**	*p*-Value *
		**N**	**%col**	**%row**	**N**	**%col**	**%row**	**N**	
**Total**		4422	100.0	48.7	4646	100.0	51.2	9068	
**Gender**									<0.001
	**Males**	3078	69.6	52.5	2790	60.1	47.6	5868	
	**Females**	1344	30.4	42.0	1856	40.0	58.0	3200	
**Age (years) mean ± std**		4422	69.6±14.2		4646	66.8±14.2		9068	<0.001
**Age class**	**(years)**								<0.001
	**19–49**	599	13.6	54.8	495	10.7	45.3	1094	
	**50–64**	1011	22.9	52.3	923	19.9	47.7	1934	
	**65–74**	1239	28.0	51.9	1149	24.7	48.1	2388	
	**75–84**	1300	29.4	45.8	1537	33.1	54.2	2837	
	**85+**	273	6.2	33.5	542	11.7	66.5	815	
**Education qualification**									<0.001
	**No qualifications/Elementary School/Middle School**	2777	62.8	46.4	3203	68.9	53.6	5980	
	**High School/Degree and more**	1645	37.2	53.3	1443	31.1	46.7	3088	
**Body Mass Index**									<0.001
	**Underweight (BMI < 18.0)**	184	4.2	37.5	307	6.6	62.5	491	
	**Normal weight (18.0 ≤ BMI < 25.0)**	2200	49.8	47.5	2432	52.4	52.5	4632	
	**Overweight (25.0 ≤ BMI < 30.0)**	1399	31.6	52.2	1282	27.6	47.8	2681	
	**Obese (BMI ≥ 30.0)**	639	14.5	50.6	625	13.5	49.5	1264	
**Self-sufficient**									<0.001
	**Complete**	2565	58.0	60.4	1679	36.1	39.6	4244	
	**Little self-sufficient**	1235	27.9	46.9	1400	30.1	53.1	2635	
	**Not self-sufficient**	622	14.1	28.4	1567	33.7	71.6	2189	
**Comorbidities**									
	**Heart disease**	1352	30.6	41.1	1941	41.8	58.9	3293	<0.001
	**Peripheral vascular diseases**	538	12.2	40.7	785	16.9	59.3	1323	<0.001
	**Cerebrovascular disease**	519	11.7	41.5	732	15.8	58.5	1251	<0.001
	**Chronic obstructive pulmonary disease**	497	11.2	38.6	790	17.0	61.4	1287	<0.001
	**Cancer**	443	10.0	40.1	663	14.3	60.0	1106	<0.001
	**Lipid metabolism's alteration**	317	7.2	51.3	301	6.5	48.7	618	0.1925
	**Neurological disease**	93	2.1	32.6	192	4.1	67.4	285	<0.001
	**Cardiovascular Risk Factors (Hypertension, Obesity, Diabetes)**	3560	80.5	50.4	3501	75.4	49.6	7061	<0.001
**Type of dialysis unit**									<0.001
	**Public hospital**	1511	34.2	44.6	1875	40.4	55.4	3386	

**Private clinic**	2911	65.8	51.2	2771	59.6	48.8	5682	
**Pre-dialysis counselling**								<0.001
**Yes**	3803	86.0	55.7	3031	65.2	44.4	6834	
**No**	618	27.7	14.0	1614	72.3	34.7	2232	

* Chi-square test or Fisher's exact for categorical variable and T-test for continuous variables.

Table S2. Association between gender (Female vs Male) and type of vascular access (AVF vs. CVC) for hemodialysis initiation adjusted for socio-demographic, clinical, and welfare variables. Univariable and multivariable logistic regression.

	**Univariable Model**				**Multivariable Model**			
**Effect**	**OR**	**95%CI**		***p*-Value**	**OR**	**95%CI**		***p*-value**
**Gender** Female vs. Male	0.66	0.60	0.72	<0.001	0.64	0.58	0.71	<0.001
**Age (years) (ref 19–49)** 50–64	0.91	0.78	1.05	0.189	0.95	0.81	1.12	0.544
65–74	0.89	0.77	1.03	0.116	1.10	0.93	1.29	0.260
75–84	0.70	0.61	0.80	<0.001	1.00	0.85	1.18	0.984
85+	0.42	0.35	0.50	<0.001	0.79	0.64	0.98	0.035
**BMI (Kg/m^2) (ref Normal weight 18.0 <= BMI <= 25.0)** Underweight BMI < 18.0	1.13	1.00	1.28	0.054	1.08	0.94	1.25	0.259
Overweight 25.0 <= BMI <= 30.0	0.66	0.55	0.80	<0.001	0.80	0.65	0.98	0.035
Obese BMI >= 30	1.21	1.10	1.33	<0.001	1.14	1.03	1.27	0.012
**Self-sufficient (ref Complete)** Little self-sufficient	0.26	0.23	0.29	<0.001	0.34	0.30	0.39	<0.001
Not self-sufficient	0.58	0.52	0.64	<0.001	0.67	0.60	0.74	<0.001
**Cardiovascular Risk Factors (Hypertension, Obesity, Diabetes)** Yes vs. No	1.35	1.22	1.49	<0.001	1.21	1.08	1.35	0.001
**Heart disease** Yes vs. No	0.61	0.56	0.67	<0.001	0.71	0.64	0.78	<0.001
**Peripheral vascular diseases** Yes vs. No	0.68	0.59	0.78	<0.001	0.84	0.73	0.98	0.025
**Cancer** Yes vs. No	0.67	0.59	0.76	<0.001	0.69	0.60	0.79	<0.001
**Chronic obstructive pulmonary disease** Yes vs. No	0.62	0.55	0.70	<0.001	0.72	0.63	0.82	<0.001
**Type of dialysis unit** Private clinic vs. Public hospital	1.30	1.20	1.42	<0.001	1.27	1.16	1.40	<0.001
**Pre-dialysis counselling** Yes vs. No	3.28	2.95	3.64	<0.001	3.35	3.00	3.74	<0.001
